# Clinician Data Scientists—Preparing for the Future of Medicine in the Digital World

**DOI:** 10.34133/2022/9832564

**Published:** 2022-09-27

**Authors:** Fulin Wang, Lin Ma, Georgina Moulton, Mai Wang, Luxia Zhang

**Affiliations:** ^1^National Institute of Health Data Science at Peking University, Beijing, China; ^2^Institute of Medical Technology, Peking University Health Science Center, Beijing, China; ^3^Peking University Health Science Center, Beijing, China; ^4^Division of Informatics, Imaging and Data Sciences, Faculty of Biology, Medicine and Health, The University of Manchester, Manchester, UK; ^5^Advanced Institute of Information Technology, Peking University, China; ^6^Institute of Nephrology, Peking University First Hospital, China

## 1. Introduction

Clinician-scientists have a unique strength in translational research and medical advances that improve the quality of care and patient outcomes. As big data analytics and advanced technologies such as artificial intelligence are being continuously applied in the healthcare scenario, it not only transforms patient care but also creates tremendous opportunities for data-driven discoveries. In a digital health era, clinician-scientists proficient in data science knowledge——that is clinician data scientists——are central to harnessing the power of big data analytics and advanced technologies in medicine.

Combining in-depth clinical knowledge with skills in data science, clinician data scientists are well prepared to identify challenges in healthcare accompanied with digital transformation, apply relevant methodologies, lead meaningful scientific studies, communicate across disciplines, and generate critical appraisal and interpretations. Equipped with interdisciplinary knowledge, the clinician data scientists will play crucial roles in guiding innovative technologies approvals and participate in making digital health policy. Clinician data scientists will often work in multidisciplinary teams: working closely with statisticians, computer scientists, and health informaticians. They will understand the clinical research question and have the appropriate awareness of the different areas to be able to translate this into digital or data protocols with others who provide the finer technical details for it to be carried out. A clinician data scientist role is essential as often data scientists or researchers who develop methodologies, for example, clinical prediction models, do not understand the challenges of translating them into clinical practice [1]. In this perspective article, we aim to highlight the core competencies required for clinician data scientists and discuss how scientific and medical communities should join efforts to nurture the emerging sector of the medical profession and prepare for the future of medicine in the digital world.

## 2. Core Competencies

It is well established that the criteria for impactful medical research include meaningful research questions that are closely informed by clinical need and in-depth understanding of medical knowledge with skills in scientific investigation and analysis [2]. And it is still the case when the engaged disciplines broadened to data science. Clinician data scientists will need to be able to closely link medicine and data science to work efficiently and to enable the discovery capability of big data in healthcare.

With this paradigm, clinician data scientists need a fundamental understanding about health data, training on epidemiology, statistics, bioinformatics, and computer science combined with an understanding of continuous healthcare improvement frameworks, socio-technical system challenges, and advanced skills in interdisciplinary communication and collaboration. Understanding the types and characteristics of heterogenic healthcare-related data, the construction of diverse databases, and data governance (data quality, data security, ethics, and law) are essential skills for clinician data scientists to consolidate achievable scientific questions and prepare for the interdisciplinary investigations [3– 5]. Meanwhile, training in the tools for statistical modeling, machine learning, nature language processing, knowledge management and data visualization will further position the clinician data scientists for success to design scientifically rigorous studies and apply up-to-date data analytics [6]. Furthermore, the ability to create close alliances with specialties from various-disciplines allows them to conduct effective research plans. Essentially, with a profound understanding of healthcare and the privilege to identify knowledge gaps in medical practice, clinician data scientists will play critical roles in the data science research projects as the domain expert. Understanding the connotation, terminology, and methodology of the data science-related disciplines will help them communicate and collaborate efficiently. In addition, clinician data scientists should be aware of methods and frameworks that enable organizational and behavioural change so as to understand how new digital interventions or a change in clinical practice will be adopted and how to engage with a range of stakeholders (including patients and citizens) to design usable digital interventions and decision support systems.

## 3. Challenges in Training

The training of clinician data scientists is challenging due to the increased complexity of data, rapid advancement of analytic techniques, and enriched medical research spectrum. Moreover, unlike the other clinician-scientists training, data science is not within the scope of conventional medical education.

At present, medical schools have started to modify their curriculum in response to the digital transformation in healthcare and the subsequent needs for data literacy in medical education. In the UK, experts suggested introducing different aspects of digital advances with relevant clinical rotations to integrate the data knowledge with clinical context and prepare additional modules for students interested in obtaining advanced skills in health data science. However, intercalating the topics into an already busy medical curriculum is under debate [4]. Currently, training in data science is obtained at post-registrar stage with funding to increase research capacity in this space available by research councils. In the US, digital health curricula are available nowadays for medical students in some universities with flexible individualized formats, although it emphasizes on improving data literacy rather than training future leaders in health data research [3]. In China, medical students at Peking University can select an introductory course for data science, and episodic, more advanced certification courses are available for residents and specialists. However, a curriculum system that focuses on the core competencies required by clinician data scientists has not yet been formed. On the other hand, a graduate degree education in health data science is available in different counties, which may help fill in the knowledge gap in data science. However, the programs usually emphasize advanced data analytics skills and are not tailored for clinicians. Overall, integrated formal training programs for clinician data scientists are scarce worldwide.

In addition, an important aspect of training clinician data scientists is to be able to meet the challenges in the delivery of such interdisciplinary education, for example, how to use the correct terminology across disciplines, or how to provide hands-on experience of working with data in EHR systems, understanding how it is recorded, and how this impacts directly on delivery of care or how digital solutions interoperate.

## 4. Addressing the Challenges

The dual identity of clinician data scientists as healthcare professionals and data scientists pose a significant challenge to the education and training programs. Identifying core competencies of clinician data scientists is essential for setting the requirement of skills [7, 8]. Accordingly, training frameworks should be designed with the flexibility to allow for adaptation to various application scenarios. Further, frequent updating of the programs to reflect new development is imperative. Finally, a close partnership of senior clinical faculties and data scientists in curriculum design is crucial to ensure that not only a program is clinician-centered but also the delivery of education is familiar. Clinicians are used to being trained in interdisciplinary teams as this is a regular feature of their job, also, they are used to simulating environments in order to hone their practical skills. This type of delivery can be integrated into data science training.

Other than self-contained data science courses, it may also be helpful to provide serials of workshops, each focusing on specific research scenarios and incorporating only necessary methodology modules across disciplines. For example, a workshop on the secondary use of electronic health records (EHR) may include EHR data ethics and governance, database structure, relevant study designs, data wrangling steps, analysis, and interpretation. In addition, succinct but application-oriented courses on advanced methodology topics (data visualization, coding, or artificial intelligence software) may help to improve data science skills.

## 5. Nurturing Future Leaders in Health Data Science

It is essential we train clinicians with data science skills, whilst at the same time training data scientists with deep technical skills so that key clinical questions are formed and can be prioritized. Clinician data scientists are critically important as they possess a deep understanding in both science and humanistic nature of medicine and are ready to identify clinically important questions that if addressed can make medical advances and assure excellence in patient care.
